# Epidemiological Features of Neurological Disorders in North Africa and the Middle East from 1990 to 2019: Results from the Global Burden of Disease Study 2019

**DOI:** 10.34172/aim.2023.13

**Published:** 2023-02-01

**Authors:** Mehdi Moradinazar, Mohmmad Shakiba, Yousef Ramazani, Sahel Kanjouri, Rozhan Shokohyzade, Sara Darvishi, Ebrahim Shakiba

**Affiliations:** ^1^Behavioral Disease Research Center, Kermanshah University of Medical Sciences, Kermanshah, Iran

**Keywords:** Burden of disease, Middle East, Neurological disorders, North Africa

## Abstract

**Background:**

The burden of neurological disorders increases with population growth and aging and nearly three-quarters of the global burden of neurological disorders has been reported in low- and middle-income countries. Therefore, this study aimed to report the epidemiological features and the burden of neurological disorders in North Africa and the Middle East (NAME) countries.

**Methods:**

The study population included 21 countries in the NAME region with a population of more than 600 million. The Global Burden of Disease (GBD) 2019 database was used. In GBD 2019, neurological disorders are classified into 7 diseases and injuries. Incidence rates, prevalence rates, death rates, disability adjusted life years (DALYs) rates by age-standardized rate (ASR) per 100000 people were measured. Also, the attributed burden to high body mass index (BMI), high fasting plasma glucose, smoking, and alcohol use were reported.

**Results:**

The highest incidence rates of neurological disorders in 2019 were in Iran 11293.27 (95% UI, 10132.62–12499.59) and Egypt 10257.33 (95% UI, 9189.37–11341.16), respectively, and the highest mortality 41.12 (95% UI, 17.68–92.44) and DALYs 1503.0 (95% UI, 853.8–2492.15) rates were in Afghanistan. In NAME region, the incidence and prevalence rate of neurological disorders increased by 0.84% (10006.37 to 10090.79) and 1.36% (33711.72 to 34170.57) respectively, while the mortality and DALYs rate decreased by 2.75% (34.11 to 33.17) and 3.92% (1438.48 to 1382.14) between 1990 and 2019. The highest decrement of the neurological disorders-related DALYs with a 10.10% decrement pertained to Afghanistan (1671.86 to 1503). The highest increment of the neurological disorders-related DALYs with a 1.89% increment pertained to Morocco (1330.69 to 1355.83). The highest attributed DALYs to alcohol use pertained to Turkey 9.8 (95% UI, 4.23–18.05). The highest attributed DALYs to high BMI 112.23 (95% UI, 29.3–285.75) and high fasting plasma glucose 100.36 (95% UI, 18.79–302.85) pertained to Qatar. The highest attributed DALYs to smoking pertained to Lebanon 106.34 (95% UI, 37.65–253.87). Most DALYs were associated with those aged 75 years and more.

**Conclusion:**

Despite progressive reduction in death due to neurological disorders in the NAME region in recent decades, there was a considerable and increasing number of people affected by different neurological disorders. As populations age, societies will face more challenges regarding prevention, detection, treatment, and rehabilitation.

## Introduction

 Neurological disorders, as the leading causes of premature deaths and transient or permanent disability, are one of the global public health challenges.^1,2^ With an aging population globally, the rate of mortality and disability caused by neurological disorders has grown substantially, and this burden is supposed to ascend during the next few decades. In fact, increased longevity and decreased fertility lead to aging populations. In 2013, around three-quarters of the global burden of neurological diseases, mental, and substance abuse disorders were reported in low- and middle-income countries.^3^ From 1990 to 2015, the absolute number of deaths caused by neurological disorders has risen by 36.7% and the disability-adjusted life-years (DALYs) increased by 7.4%.^4^ In 2016, globally, neurological disorders with 276 million (95% uncertainty intervals [UI] 247-308) DALYs were found as the leading cause of DALYs and with 9.0 million (95% UI 8.8-9.4) deaths were the second leading cause of death.^1^ Also, neurological disorders impose considerable costs on societies, patients, and the health systems.

 In the North Africa and the Middle East (NAME) region, most health determinants are similar; however, there is a significant discrepancy in health and disease index among these countries.^5^ Neurological disorders are one of these diseases, which vary in prevalence, incidence, death, and DALY across these countries. Therefore, national health policymakers should be informed about their society’s health compared with other societies with a similar socioeconomic status. They can also utilize other countries’ experiences in improving their health care. Global Burden of Disease (GBD) studies provide a chance to compare societies and explain the pattern of diseases.^4^

 Health policymakers require actual and creditable up-to-date reports of neurological disorders across various countries and populations. Such estimates set priorities and help with tailored cost-benefit health interventions and resource allocation. Epidemiological surveys play an important role in the recognition of the outbreak, patterns, risk factors, and etiology of diseases. Therefore, the aim of this study was to determine the epidemiological burden of neurological disorders in the NAME countries to tailor beneficial policies for decreasing the incidence, prevalence, death, and complications of neurological disorders in this region.

## Materials and Methods

###  Geographical Location and Population 

 The NAME region, with a population of more than 600 million (608 713 600), consists of 21 countries including Afghanistan, Algeria, Bahrain, Egypt, Iran, Iraq, Jordan, Kuwait, Lebanon, Libya, Morocco, Palestine, Oman, Qatar, Saudi Arabia, Sudan, Syria, Tunisia, Turkey, United Arab Emirates (UAE), and Yemen, which were compared in terms of age, sex, and risk factors for neurological disorders.

###  Data Collection and Quality Control

 The GBD 2019 database was used in the current study. The data of GBD estimates the deaths and disability due to 369 diseases and injuries in terms of location, age, and sex for 204 countries and regions (https://vizhub.healthdata.org/).^6^ In this system, modeling for each country is according to data availability and quality. The GBD 2019 determined the causes of mortality coded as neurological disorders using verbal autopsy reports (VAR) and vital registration systems (VRS). The data are analyzed with the Cause of Death Ensemble model (CODEm). The CODEm, as a good systematized instrument, applies different models on the same data and selects a set of models that best reflect all the input data. The prevalence and incidence of neurological disorders rates were estimated using the DisMod-MR 2.1 (disease-model Bayesian meta-regression) modeling software. DisMod-MR 2.1, as a Bayesian geospatial disease modeling tool, utilizes data on different disease parameters, the epidemiological associations between these parameters, and geospatial communications to approximate the prevalence and incidence. First, it is run on data from all countries, which makes a primary global fit, and calculates coefficients for predictors and the adjustments for alternative study characteristics. The global fit adjusted by the random effect values for each of the seven GBD super regions, the coefficients on gender, and country predictors, is transferred as data to a model for each of the seven GBD regions together with the input data for that geography. These steps are replicated from seven GBD regions to 21 regions, 204 countries, and where applicable, to subnational units.^7^

 Furthermore, the same method was used for each place and time in all countries of the NAME region, to achieve reliable and valid comparisons across various locations and years.

###  Deﬁnition of Neurological Diseases

 In GBD 2019, neurological disorders incorporate 7 diseases and injuries including Alzheimer’s and other dementias, Parkinson’s, epilepsy, multiple sclerosis, motor neuron disease, headache disorders, and other neurological disorders (excluding stroke, meningitis, tetanus, brain cancer, and trauma).

 The GBD study evaluated the different risk factors attributable to neurological disorders-related DALYs consisting of high body mass index (BMI), high fasting plasma glucose, smoking, and alcohol use.^8^ Further information is available at http://vizhub.healthdata.org/gbd-compare/.

###  DALY Calculation

 The rates in GBD are standardized based on the total world population. DALYs index was calculated to compare different countries. DALYs are the sum of the years of life lost (YLLs) and the years lived with disability (YLDs).^9,10^ YLLs are counted as the product of the estimated number of deaths and a standard life expectancy at the age of death. YLDs are computed by multiplying the prevalence of individual consequences of the disease, by their corresponding disability weights, which quantify the severity of consequences as a number between 0 (full health) and 1 (death). Details of data sources and estimation methods have been published before.^6,11^ Also, the age-standardized rate (ASR), age-standardized incidence rate, age-standardized prevalence rate, and age-standardized death rate in 100 000 people were reported.^12,13^

 All data were extracted from GBD (https://vizhub.healthdata.org/). In general, the external validity of GBD was evaluated by performing cross-validation on a limited number of sequelae due to the computational time and complexity of this analysis. Then, we analyzed the data based on the study’s objectives e.g. age groups, risk factors, gender etc. Also, all estimates were reported with 95% UI. All analyses and figures were undertaken using Microsoft Office Excel 2016.

## Results

 In the NAME region, the incidence rate of neurological disorders increased by 0.84% (10006.37-10090.79), the prevalence rate increased by 1.36% (33711.72-34170.57), the mortality rate decreased by 2.75% (34.11-33.17), and the DALY rate decreased by 3.92% (1438.48-1382.14) between 1990 and 2019.

 In terms of ASR, the incidence rate of neurological disorders increased from 1990 to 2019 in the countries of the NAME region except for Algeria, Bahrain, Kuwait, Oman, Palestine, Qatar, Saudi Arabia, Sudan, and the United Arab Emirates. The highest incidence rates in 1990 pertained to the Islamic Republic of Iran 10802.77 (95% UI, 9740.21–11910) and Egypt 10148.8 (95% UI, 9060.23–11273.99), and the countries of Turkey 9507.57 (95% UI, 8483.29–10571.1) and Saudi Arabia 9656.09 (95% UI, 8605.59–10722.53) had the lowest incidence rate of neurological disorders, respectively. But in 2019, the Islamic Republic of Iran 11293.27 (95% UI, 10132.62–12499.59) and Egypt 10257.33 (95% UI, 9189.37–11341.16) continued to have the highest incidence rates of neurological disorders, while the lowest rate was found in Saudi Arabia 9582.12 (95% UI, 8516.51–10684.85) and Turkey 9588.75 (95% UI, 8564.72-10698.3).

 Furthermore, the average global incidence rate of neurological disorders slightly decreased from 10266.11 (95% UI, 9263.78–11327.09) to 10259.5 (95% UI, 9223.2–11324.16) between 1990 and 2019. In the countries of the NAME region except for Iran, the incidence rate of neurological disorders was found to be lower than the average global incidence rates in 2019. The DALYs rate of neurological disorders in the countries of the NAME region was higher than the global average in 2019. In the countries of the NAME region except for Jordan and Kuwait, mortality rates were found to be higher than the global average in 2019. However, in NAME region, the prevalence, mortality, and DALYs rates were reported to be higher than the global average in 2019.

 The highest mortality rates in 1990 pertained to Afghanistan 44.05 (95% UI, 19.77–97.51), and the lowest mortality rates were found in Egypt 30.9 (95% UI, 12.57–73.56). But in 2019, the lowest rate belonged to Kuwait 29.95 (95% UI, 11.35–68.98). The highest DALYs rates in 1990 were found in Afghanistan 1671.86 (95% UI, 1006.8–677.72) and Turkey 1618.99 (95% UI, 979.62–2562.35), respectively, and the Syrian Arab Republic 1308.92 (95% UI, 702.93–2206.65) had the lowest DALYs rate of neurological disorders. But in 2019, the highest DALYs rate pertained to Afghanistan 1503.0 (95% UI, 853.8-2492.15), and the lowest DALYs rates were found in Jordan 1291.82 (95% UI, 687.68–2194.39) and Kuwait 1294.92 (95% UI, 693.83–2173.19), respectively (Table 1).

**Table 1:** Comparison of Neurological Disorders Burden in the NAME Countries

**Country**	**Year**	**Incidence Rate of Neurological Disorders by ASR**	**Prevalence Rate of Neurological Disorders by ASR**	**Death Rate of Neurological Disorders by ASR**	**DALYs Rate of Neurological Disorders by ASR**
Global	1990	10266.11(9263.78-11327.09)	33313.53(30763.88-35884.2)	30.3(13.67-67.71)	1264.17(740.27-2040.8)
	2019	10259.5(9223.2-11324.16)	33451.93(30870.74-36082.04)	30.68(13.83-66.32)	1253.56(719.7-2039.81)
NAME	1990	10006.37(8926-11068.08)	33711.72(30876.29-36594.29)	34.11(14.73-80.09)	1438.48(831.35-2364.85)
	2019	10090.79(9024.07-11159.26)	34170.57(31389.49-37068.51)	33.17(14.25-73.94)	1382.14(776.61-2273.81)
Afghanistan	1990	9897.72(8812.36-10960.33)	33547.28(30576.69-36590.08)	44.05(19.77-97.51)	1671.86(1006.8-2677.72)
	2019	9901.89(8816.45-10966.09)	33401.51(30465.41-36481)	41.12(17.68-92.44)	1503(853.8-2492.15)
Algeria	1990	9913.71(8826.14-11008.15)	33526.8(30482.46-36559.76)	38.26(16.41-90.6)	1489.82(852.43-2446.54)
	2019	9906.77(8817.67-10981.69)	33570.12(30564.25-36622.75)	34.24(13.65-81.72)	1365.12(738.51-2292.05)
Bahrain	1990	9911.95(8819.31-11020.67)	33135.13(30194.43-36218.06)	38.78(17.01-88.64)	1508.89(891.21-2415.66)
	2019	9902.56(8826.12-10988.28)	33112.7(30197.4-36147.05)	36.04(15.19-80.67)	1387.65(768.62-2266.38)
Egypt	1990	10148.8(9060.23-11273.99)	34746.07(31587.03-37944.21)	30.9(12.57-73.56)	1324.67(706.76-2240.15)
	2019	10257.33(9189.37-11341.16)	35726.9(32855.16-38512.6)	31.24(12.79-70.09)	1336.25(709.79-2284.83)
Islamic Republic of Iran	1990	10802.77(9740.21-11910)	36380.95(33701.34-39038.77)	32.56(13.28-76.36)	1454.4(829.04-2403.79)
	2019	11293.27(10132.62-12499.59)	38297.19(35449.74-41091.4)	32.1(13.23-73.93)	1394.23(785.78-2300.94)
Iraq	1990	9902.38(8817.24-10991.94)	33433.28(30414.03-36422.88)	32.05(12.99-76.9)	1355.48(740.69-2270.59)
	2019	9902.57(8818.84-10985.86)	33513.59(30526.39-36599.69)	32.45(13.34-72)	1331.77(715.6-2250.9)
Jordan	1990	9884.59(8806.96-10983.08)	33341.32(30395.71-36423.08)	33.46(14.04-77.66)	1355.61(760.12-2254.54)
	2019	9888.49(8805.23-10979.73)	33366.2(30403.82-36446.09)	30.3(12.43-67.56)	1291.82(687.68-2194.39)
Kuwait	1990	9902.3(8804.95-10973.23)	33019.24(30088.03-36145.44)	32.28(13.07-72.26)	1352.61(754.43-2196.75)
	2019	9888.63(8803.29-10919.93)	33271.99(30301.23-36326.14)	29.95(11.35-68.98)	1294.92(693.83-2173.19)
Lebanon	1990	9897.19(8792.81-10999.32)	33554.27(30604.46-36661.28)	34.74(13.87-81.06)	1396.92(770.79-2336.79)
	2019	9912.93(8824.46-10995.56)	33723.81(30708.5-36796.55)	33.02(12.65-78.45)	1349.31(721.7-2279.85)
Libya	1990	9899.78(8813.17-11000.85)	33265.26(30358.74-36313.83)	36.48(14.52-84.47)	1420.67(799.21-2336.77)
	2019	9902.44(8808.55-10993.8)	33496.45(30469.04-36534.72)	35.77(14.29-80.84)	1378.2(759.17-2303.78)
Morocco	1990	9903.54(8809.53-10989.52)	33463.35(30392.21-36531.33)	31.03(11.88-78.29)	1330.69(718.96-2240.55)
	2019	9904.17(8826.3-10974.42)	33537.02(30530.41-36612.59)	33.11(13.63-77.96)	1355.83(744.58-2284.87)
Oman	1990	9867.95(8802.71-10950.48)	32812.85(29892.95-35866.63)	37.31(14.24-86.04)	1327.72(722.95-2262.27)
	2019	9864.6(8795.8-10966.59)	32834.25(29837.34-35871.31)	39.08(17.07-90.09)	1340.63(758.23-2231.21)
Palestine	1990	9911.71(8820.66-10986.15)	33553.11(30608.76-36614.27)	36.9(15.61-85.9)	1450.2(805.31-2386.75)
	2019	9904.42(8818.61-11008.6)	33527.94(30565.05-36597.73)	34.46(14.95-80.34)	1376.01(773.77-2255.82)
Qatar	1990	9826.62(8765.05-10927.08)	32464.32(29518.98-35464.97)	36.31(16.13-84.21)	1374.59(798.24-2287.23)
	2019	9777.42(8710.91-10885.33)	32245.08(29313.37-35220.66)	40.27(18.1-90.26)	1344.15(754.06-2196.03)
Saudi Arabia	1990	9656.09(8605.59-10722.53)	32038.56(29181.06-34881.34)	37.05(16.1-82.99)	1426.77(797.89-2323.23)
	2019	9582.12(8516.51-10684.85)	31719.53(28912.01-34577.54)	36.34(16.67-78.13)	1428.39(794.93-2355.59)
Sudan	1990	9892.18(8802.24-10986.95)	33389.37(30454.7-36493.93)	33.71(14.41-78.37)	1457.6(821.2-2437.76)
	2019	9879.28(8797.88-10946.46)	33434.1(30397.06-36506.11)	31.38(13.01-72.48)	1353.83(761.54-2268.16)
Syrian Arab Republic	1990	9891.04(8813.8-10960.95)	33366.68(30355.39-36485.28)	31.49(12.19-75.97)	1308.92(702.93-2206.65)
	2019	9914.99(8823.25-10985.65)	33730.33(30733.51-36791.65)	33.03(13.11-78.28)	1327.06(692.36-2305.8)
Tunisia	1990	9895.22(8801.3-10990.69)	33445.69(30462.41-36501.93)	33.42(12.8-80.03)	1361.56(758.07-2289.81)
	2019	9908.38(8818.41-10995.92)	33620.26(30667.97-36658.69)	32.72(12.43-75.92)	1332.58(699.8-2234.86)
Turkey	1990	9507.57(8483.29-10571.1)	31653.1(28894.59-34571.48)	37.05(16.79-79.13)	1618.99(979.62-2562.35)
	2019	9588.75(8564.72-10698.3)	31966.21(29201.81-34866.92)	34.48(15.47-78.34)	1477.44(862.48-2368.43)
United Arab Emirates	1990	9869.68(8810.27-10963.81)	32676.07(29672.03-35718.39)	38.26(17.8-85.58)	1530.62(907.12-2442.63)
	2019	9865.57(8796.42-10962.29)	32631.28(29615.67-35673.47)	35.56(16.78-76.6)	1432.05(843.2-2254.35)
Yemen	1990	9894.37(8804.99-10967.52)	33389.57(30398.46-36465.34)	31.54(12.4-75.98)	1361.07(724.79-2337.66)
	2019	9895.36(8807.41-10988.43)	33439.19(30457.56-36527.79)	32.1(12.48-77.43)	1324.53(723.36-2244.2)

**Figure 1:**
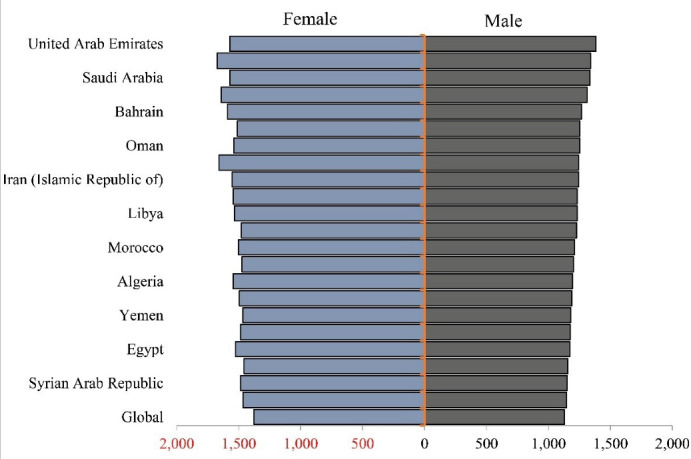
DALY Rates of Neurological Disorders According to ASR in the NAME Region in 2019

 The DALYs rates in the NAME region countries among women and men were higher than the global average in terms of population ratio in 2019. In terms of ASR, Afghani, Qatari, and Turkish women had the highest DALYs rate of neurological disorders in the region. Men in the United Arab Emirates, Afghanistan and Saudi Arabia had the highest DALYs rates according to ASR in the region. In general, women had higher neurological disorders-related DALYs rates than men in all countries (Figure 1).

**Figure 2:**
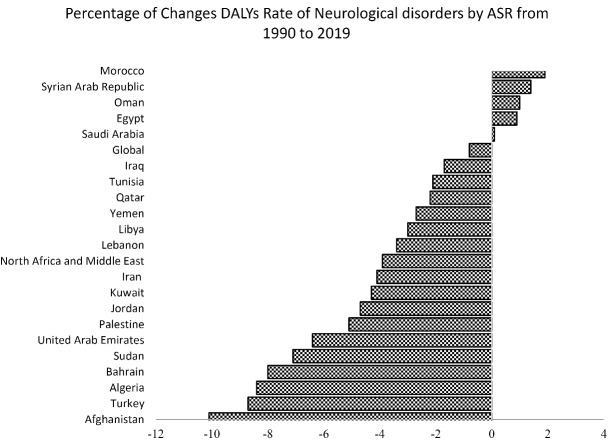
Percentage Changes in the DALYs Rate According to ASR from 1990 to 2019

 Between 1990 and 2019, the highest decrement of the neurological disorders-related DALYs with a 10.10% decrement pertained to Afghanistan (1671.86 to 1503). The highest increment of the neurological disorders-related DALYs with a 1.89% increment belonged to Morocco (1330.69 to 1355.83) (Figure 2).

**Figure 3:**
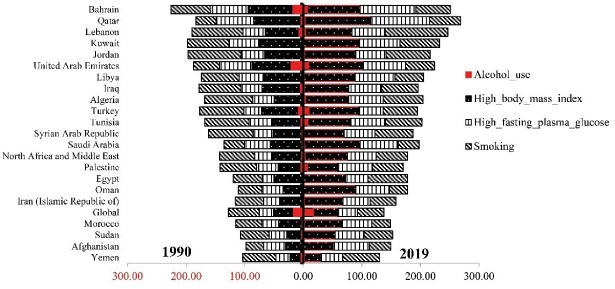
DALYs Rate of Neurological Disorders Attributed to Smoking, High Body Mass Index, High Fasting Plasma Glucose, and Alcohol Use

**Figure 4:**
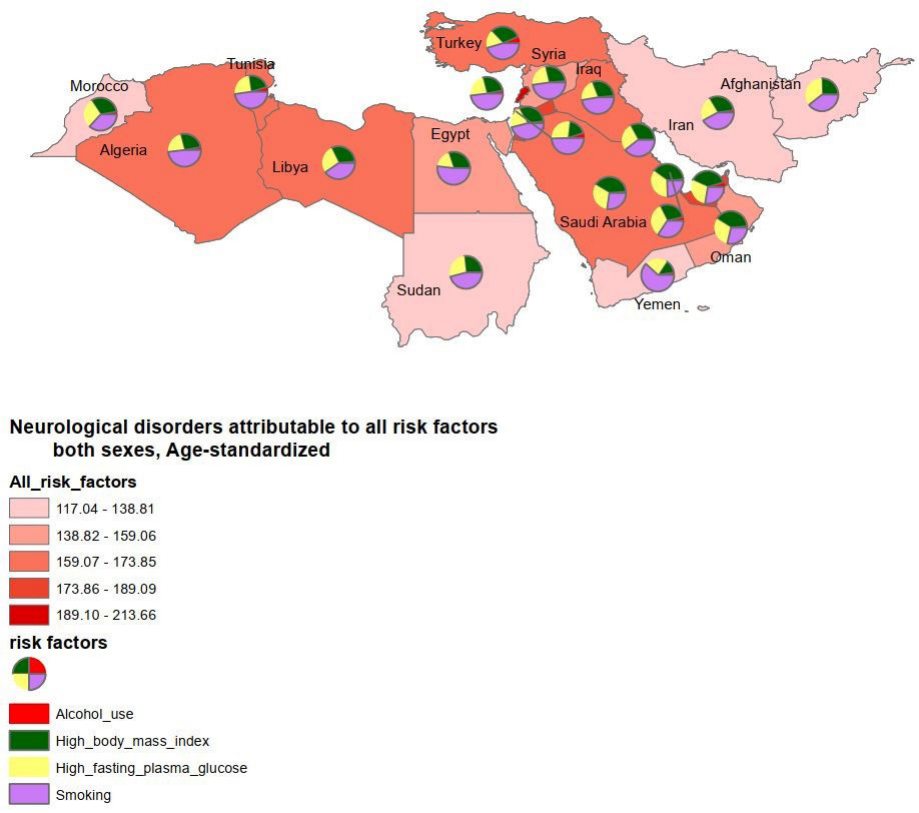
DALYs Rate of Neurological Disorders Attributed to Smoking, High Body Mass Index, High Fasting Plasma Glucose, and Alcohol Use in 2019

 In 2019, the highest burden attributed to alcohol use belonged to Turkey 9.8 (95% UI, 4.23–18.05), while Sudan 0.15 (95% UI, 0.04–0.39) had the lowest DALYs due to alcohol use. The highest burden attributed to high BMI 112.23 (95% UI, 29.3–285.75) and high fasting plasma glucose 100.36 (95% UI, 18.79–302.85) pertained to Qatar, whereas Yemen had the lowest DALYs rates due to high BMI 27.67 (95% UI, 6.2–78.56) and high fasting plasma glucose 37.64 (95% UI, 6.28–123.82). The highest burden attributed to smoking was found in Lebanon 106.34 (95% UI, 37.65–253.87) while the lowest belonged to Oman 30.9 (95% UI, 8.53-76.91) in 2019 (Figures 3 and 4). ArcGIS 10.7.1 was used to prepare Figure 4.

**Figure 5:**
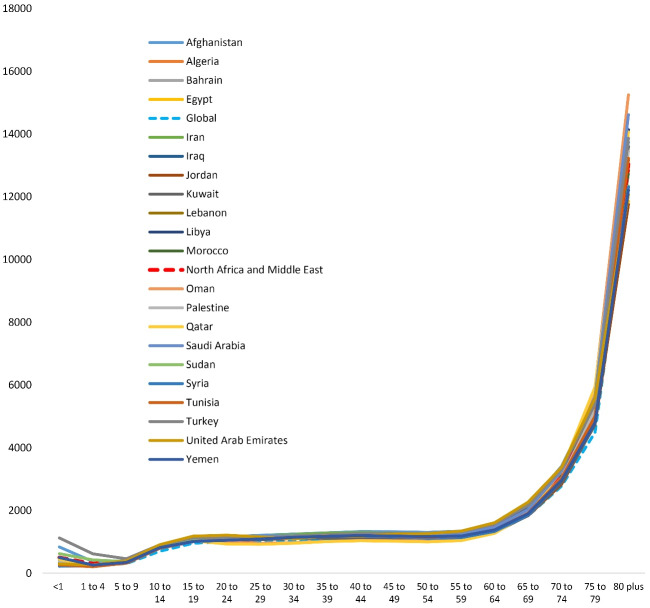
The Age Trend of the DALYs Rate of Neurological Disorders in 2019

 Although neurological disorders-related DALYs in the countries of the NAME region were higher than the global average, most DALYs, similar to the global trend, were associated with those aged 75 years and above (Figure 5).

**Figure 6:**
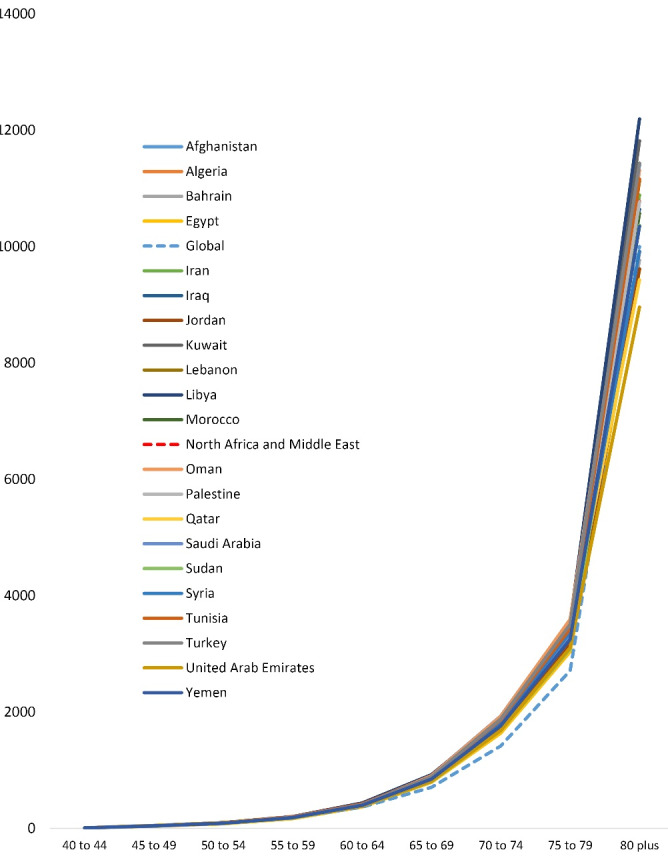
The Age Trend of the DALYs Rate of Alzheimer’s and Other Dementias in 2019

 DALYs of Alzheimer’s disease and other dementias in the NAME countries were higher than the global average, with most DALYs, similar to the global pattern, pertaining to those aged 75 years and above (Figure 6).

**Figure 7:**
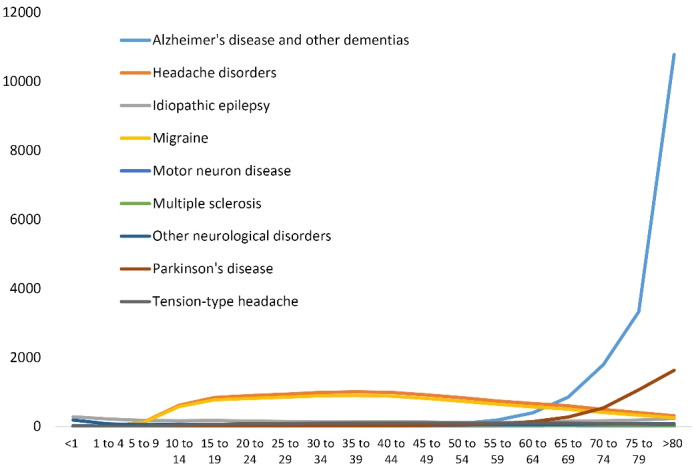
The Age Trend of the DALYs Rate of Alzheimer’s and Other Dementias, Parkinson, Epilepsy, Multiple Sclerosis, and Motor Neuron Diseases in 2019

 The DALYs rate for Alzheimer’s disease and other dementias, Parkinson’s disease, and multiple sclerosis were 3325.9, 1064.1, and 31.7, respectively among the age group of 75 to 79 years in 2019 (Figure 7).

## Discussion

 Results from the present study revealed that between 1990 and 2019, the incidence and prevalence rate of neurological disorders increased by 0.84% and 1.36%, respectively, while the mortality and DALYs rate decreased by 2.75% and 3.92%.

 The findings of the present study demonstrated that the incidence rate of neurological disorders has increased from 1990 to 2019 in all countries in the NAME region except Algeria, Bahrain, Kuwait, Oman, Palestine, Qatar, Saudi Arabia, Sudan, and the United Arab Emirates. Furthermore, previous evidence indicated that the incidence and burden of neurological diseases have increased over the years,^14-16^ while their prevalence decreased. This may be due to the aging societies, the development of therapeutic facilities and survival, and the increasing number of neurologists.^17,18^ The countries of the Islamic Republic of Iran, Egypt, and Morocco had the highest, and Saudi Arabia and Turkey had the lowest neurological disorders incidence rates by ASR among the NAME countries in 1990. In 2019, the Islamic Republic of Iran, Egypt, and Morocco continued to have the highest incidence rate, and Turkey and Saudi Arabia continued to have the lowest incidence rate.

 An examination of the trend of neurological disorders incidence rate by ASR from 1990 to 2019 illustrated that the incidence rate in the NAME region was lower than the global average, but in the Islamic Republic of Iran, it was greater than the world average between 1990 to 2019. From 1990 to 2019, the incidence rate of neurological disease increased from 7.5 million to more than 12 million, and the incidence rate elevated to 1400 per 100 000 population in the Islamic Republic of Iran.^19^ This phenomenon may be caused by the impaired health systems and the consequences of almost one decade of the Iran–Iraq War, dispersion and displacement, tensions, destruction of health care foundations, and nowadays, sanctions or political conflicts.

 As mentioned above, Saudi Arabia had the lowest incidence rate of neurological disorders. Actually, local population-based studies in Saudi Arabia demonstrated that the prevalence ratio of stroke is about 1.78.^20^ Also, the prevalence of headaches in Saudi Arabia was found to be significantly low (8-12%) compared to other countries in the region (for example, 72.5% for Qatar and 83.6% for Oman).^21^ Also, Al-Khamis conducted a study to assess the pattern of neurological disorders in the neurology outpatient clinics at the tertiary care level, reporting that the prevalence of epilepsy and seizure disorders, headaches, stroke, and multiple sclerosis were 37.71%, 15.51%, 9.29%, and 7.41% respectively.^22^

 There has been a decreasing trend in the DALY and death rates caused by neurological disorders in the NAME region. The DALY rates of neurological disorders have decreased from 1990 to 2019 in most countries of the NAME region, but DALYs in Egypt, Oman, Saudi Arabia, and the Syrian Arab Republic increased slowly. DALY and death rates of neurological disorders in the NAME region were higher than the global average, which might be due to the fact that in the NAME region, health infrastructures have not had enough funding and resources; so the screening programs, early detection, appropriate treatment, and medical standards were not effectively applied.^23^ Therefore, it is suggested that easy and economic screening tests should be used to decrease death and DALY rates caused by neurological disorders in these countries; it is also suggested to develop a healthy lifestyle by educational programs.

 The highest death and DALY rates in 1990 were found in Afghanistan, and this country continued to have the highest death and DALYs rates in 2019. In 2016, Afghanistan had DALY rates of neurological disorders exceeding 7000 DALYs per 100 000 population by ASR.^7^ Afghanistan had the highest prevalence rate of tension-type headache by ASR in the Eastern Mediterranean Region.^24^ This might be due to persistent political tensions, civil war, and terrorism in Afghanistan, which have damaged the population’s health and the healthcare infrastructures.

 Globally, there were notable increases in stroke-related DALYs attributed to alcohol use ( > 32% increase), smoking ( > 10% increase), high BMI ( > 46% increase), and high fasting plasma glucose (~44% increase).^25^ In 2019, Turkey had the highest burden attributed to alcohol use in the NAME region. The highest burden attributed to high BMI and high fasting plasma glucose pertained to Qatar. The highest burden attributed to smoking belonged to Lebanon in 2019. Our findings showed that the highest impact of risk factors in neurological disorders-related DALY index in Iran and the NAME region was attributed to high BMI. According to previous evidence, obesity is a major risk factor for the onset and progression of some neurological disorders. Obesity-induced dyslipidemia, metabolic dysfunction, and inflammation affect the central nervous system (CNS). Parkinson’s disease and Alzheimer’s disease could be started by different metabolic changes caused by obesity and damage to the CNS. These metabolic processes change the synaptic flexibility of the neurons and result in neural death, and have detrimental effects on the normal physiology of the CNS. Therefore, a healthy diet and exercise, as an effective non-invasive strategy, can counteract neurological disorders.^26^ From 1990 to 2019, the countries of Afghanistan and Turkey had the highest percentage changes in the DALYs rate, and Saudi Arabia reported the lowest percentage changes in the DALYs rate of neurological disorders. The dominant risk factor in Turkey and Saudi Arabia was high BMI, and the dominant risk factor in Afghanistan was high fasting plasma glucose.

 In NAME, the highest rate of reduction in death pertained to Algeria, which moved from the 3rd to the tenth rank (death rate by ASR in Algeria, from 38.26 in 1990 to 34.24 in 2019). The highest rate of increase in death was observed in Qatar, which moved from the tenth to the second rank (death rate by ASR in Qatar, from 36.31 in 1990 to 40.27 in 2019). Also, Afghanistan, Qatar, and Oman had the highest death rate caused by neurological disorders despite decreasing rates of death from 1990 to 2019, which shows the importance of neurological disorders in these countries.

 We found that the highest DALY rates of neurological disorders were reported to be associated with the age of 75 years and above. The prevalence of most neurological diseases increases sharply with age.^27^ Parkinson’s disease, stroke, Alzheimer’s disease and other dementias are the three most burdensome neurological disorders in the world which develop around the age 60 or older.^28-31^ This may be a strong reason for the high rate of DALYs in the age groups of 75 years and above.

 All limitations of the GBD study have been characterized elsewhere^11^ and completely apply to this study. Moreover, we could not present the burden of all neurological disorders in detail, because these will be the aims of separate studies. In addition, some neurological disorders (e.g. restless leg syndrome and peripheral neuropathy) were not assessed by GBD. Another major limitation of this study is lack of information on other risk factors. Furthermore, the quality of the data collection system is different across countries, and the comparison of countries may be ambiguous. In July 2011, South Sudan gained independence and separated from Sudan but in this study, data for Sudan and South Sudan are reported as one country. Likewise, the uncertainty intervals do not account for several sources of bias including measurement bias, selection bias due to missing data, and model specification bias. Since GBD estimates are updated annually, the present limitations should be addressed. This study has some strengths; for example, it compared the data of countries that have the same information registration system. In addition, this study compared various indicators across different countries, which presented as collective information in GBD; so it can recognize the risk factors and the strengths and weaknesses of various countries.

 In conclusion,despite death reduction in the NAME region in recent decades, there was an increasing number of people suffering from different neurological disorders. As populations age, societies will face more challenges regarding prevention, detection, treatment, and rehabilitation. In the end, the health policymakers can use the findings of the present study to improve their health planning and resource allocation, and decrease the mortality and disability caused by neurological disorders.
